# Persistently High Rates of Abdominal Computed Tomography Imaging Among Patients With Inflammatory Bowel Disease Who Present to the Emergency Department

**DOI:** 10.1093/jcag/gwac029

**Published:** 2022-10-28

**Authors:** Rana Kandel, Maria Merlano,, Pearl Tan, Gurmun Brar, Ranjeeta Mallick, Blair Macdonald, Catherine Dubé, Sanjay Murthy, Ian Stiell, Jeffery D McCurdy

**Affiliations:** Faculty of Medicine, University of Ottawa, Ottawa, Ontario, Canada; Ottawa Hospital Research Institute, University of Ottawa, Ottawa, Ontario, Canada; Faculty of Medicine, University of Ottawa, Ottawa, Ontario, Canada; Faculty of Medicine, University of Ottawa, Ottawa, Ontario, Canada; Faculty of Medicine, University of Ottawa, Ottawa, Ontario, Canada; Ottawa Hospital Research Institute, University of Ottawa, Ottawa, Ontario, Canada; Ottawa Hospital Research Institute, University of Ottawa, Ottawa, Ontario, Canada; Department of Medical Imaging, The Ottawa Hospital, Ottawa, Ontario, Canada; Faculty of Medicine, University of Ottawa, Ottawa, Ontario, Canada; Ottawa Hospital Research Institute, University of Ottawa, Ottawa, Ontario, Canada; Department of Medicine, Division of Gastroenterology, The Ottawa Hospital, Ottawa, Ontario, Canada; Faculty of Medicine, University of Ottawa, Ottawa, Ontario, Canada; Ottawa Hospital Research Institute, University of Ottawa, Ottawa, Ontario, Canada; Department of Medicine, Division of Gastroenterology, The Ottawa Hospital, Ottawa, Ontario, Canada; Faculty of Medicine, University of Ottawa, Ottawa, Ontario, Canada; Ottawa Hospital Research Institute, University of Ottawa, Ottawa, Ontario, Canada; Department of Emergency Medicine, The Ottawa Hospital, Ottawa, Ontario, Canada; Faculty of Medicine, University of Ottawa, Ottawa, Ontario, Canada; Ottawa Hospital Research Institute, University of Ottawa, Ottawa, Ontario, Canada; Department of Medicine, Division of Gastroenterology, The Ottawa Hospital, Ottawa, Ontario, Canada

**Keywords:** *Computed tomography*, *Emergency department*, *Encounter*, *Guidelines*, *Imagings*, *Inflammatory bowel disease*

## Abstract

**Background:**

Recent guidelines recommended judicious use of abdominal computed tomography (CT) in the emergency department (ED) for inflammatory bowel disease. Trends in CT utilization over the last decade, including since the implementation of these guidelines, remain unknown.

**Methods:**

We performed a single-centre, retrospective study between 2009 and 2018 to assess trends in CT utilization within 72 h of an ED encounter. Changes in the annual rates of CT imaging among adults with IBD were estimated by Poisson regression and CT findings by Cochran-Armitage or Cochran-Mantel Haenszel tests.

**Results:**

A total of 3000 abdominal CT studies were performed among 14,783 ED encounters. CT utilization increased annually by 2.7% in Crohn’s disease (CD) (95% confidence interval [CI], 1.2 to 4.3; *P* = 0.0004), 4.2% in ulcerative colitis (UC) (95% CI, 1.7 to 6.7; *P* = 0.0009) and 6.3% in IBD unclassifiable (95% CI, 2.5 to 10.0; *P* = 0.0011). Among encounters with gastrointestinal symptoms, 60% with CD and 33% with UC underwent CT imaging in the final year of the study. Urgent CT findings (obstruction, phlegmon, abscess or perforation) and urgent penetrating findings alone (phlegmon, abscess or perforation) comprised 34% and 11% of CD findings, and 25% and 6% of UC findings, respectively. The CT findings remained stable overtime for both CD (*P* = 0.13) and UC (*P* = 0.17).

**Conclusion:**

Our study demonstrated persistently high rates of CT utilization among patients with IBD who presented to the ED over the last decade. Approximately one third of scans demonstrated urgent findings, with a minority demonstrating urgent penetrating findings. Future studies should aim to identify patients in whom CT imaging is most appropriate.

## INTRODUCTION

Cross-sectional imaging of the abdomen can be a useful diagnostic tool in inflammatory bowel disease (IBD). It complements endoscopy by providing information on the extent and severity of luminal disease and can detect extraluminal complications such as abscesses and fistulas. Furthermore, it can aid in the detection of pathologies that may mimic IBD. Computed tomography (CT) imaging is widely available, noninvasive, acquires images rapidly, and has high spatial resolution, all of which make it a favorable diagnostic tool in the emergency department (ED) (1).

CT imaging, however, may adversely impact patient experience in the ED by increasing wait times, affecting patient flow, and limiting access to CT imaging for others. The most notable concern for CT imaging is the risk of cancer from repeated exposure to ionizing radiation (2–4). Modeling studies suggest an increased risk of cancer past a threshold of 50 mSv of ionizing radiation (5,6). This equates to approximately four abdominal CT scans using conventional imaging protocols of approximately 15 mSv (2–4). However, doses as low as 10 mSv may impart a 1:1000 attributable lifetime risk of solid organ cancers or leukemia (7,8). These concerns are particularly relevant in IBD, where the onset of disease often occurs at a young age, and the relapsing-remitting course predisposes patients to repeated imaging tests over a lifetime (6,9–12). The risk of malignancy is further compounded by intestinal inflammation (13–15), and the need for immunosuppressive therapies, both of which increase the inherent risk of malignancy (16–18).

Judicious use of CT imaging has therefore been recommended, along with radiation-free alternatives when available (19–26). The 2016 Canadian Choosing Wisely campaign advised CT imaging in the acute setting only when there is high suspicion of complications (abscess, perforation or obstruction), or when symptoms suggest a non-IBD etiology (19). Moreover, the European Crohn’s and Colitis Organization (ECCO), the European Society of Gastrointestinal and Abdominal Radiology (ESGAR), and the British Society of Gastroenterology advise consideration of MR enterography and ultrasound as alternatives (20,21,26).

Although historical studies have demonstrated high rates of CT utilization among patients with IBD presenting to the ED (27–29), recent rates of CT use among this population remain unknown. Therefore, the overarching aim of our study was to determine the proportion of patients with IBD who underwent CT imaging following an ED encounter over the last decade, and to determine whether rates of use changed over time. We hypothesized that the rates of ED-related CT imaging have declined due to greater awareness of the potential harms of repeated radiation exposure with this imaging modality.

## METHODS

### Study Design and Cohort Generation

We performed a retrospective single-centre observational study at The Ottawa Hospital, a tertiary care centre in Ottawa, Canada with approximately 1200 inpatient beds and 60,000 acute care admissions per year. The study was approved by our institutional research ethics board.

All ED encounters involving adults (≥17 years) with a pre-existing diagnosis of IBD were identified by our institutional database between January 1, 2009 and December 31, 2018. A look-back period of up to 1999 was used to determine a diagnosis of IBD based on a minimum of two administrative claims using International Classification of Diseases (ICD) codes (K50* for Crohn’s disease [CD], and K51* for ulcerative colitis [UC]). Patients were required to have at least one IBD claim before the index ED encounter to capture patients with a pre-existing diagnosis of IBD. Patients with claims for both CD and UC were considered IBD-type unclassifiable (IBDU) until the specific IBD subtype was clarified by manual chart review in the second part of our study.

Abdominal and/or pelvic CT utilization within 72 h of ED presentation was determined using local imaging codes (Supplementary Table 1). A cut-off of 72 h was chosen to avoid missed scans relating to delays in access, next day imaging for patients who were admitted overnight, and imaging arranged by the ED encounter to be performed early post-discharge.

A manual chart review was subsequently performed to determine the dominant finding of each CT study. For this part, we included the most relevant patient encounters where there may be greater diagnostic uncertainty. Encounters were excluded if: (a) patients presented without gastrointestinal symptoms, (b) underwent abdominal surgery within 1 month before the encounter, (c) underwent CT imaging to restage a known cancer or pre-existing abscess or (d) left the hospital without being seen by a physician.

### Objectives, Definitions and Data Collection

Our primary objective was to determine the proportion of IBD-related ED encounters leading to abdominopelvic CT imaging within 72 h of presentation. Secondary objectives were to determine (a) annual trends in CT utilization, (b) types of CT scan findings, (c) proportion of scans with urgent IBD-related findings and (d) to assess for changes in urgent IBD-related findings over time.

#### CT Finding Characterization

The chart review was performed by M.M. and R.K. with oversight from the senior investigator (J.M.). To ensure reliability, all CT reports with unclear findings were reviewed with the senior investigator (J.M.). Furthermore, a random audit of 50 reports from each reviewer was performed by the senior investigator (J.M.) to ensure consistency. CT findings were classified as normal, IBD-related and IBD-unrelated (Table 1). IBD-related findings were subdivided based on the Montreal Classification Hierarchy: penetrating (fistula, phlegmon, abscess or perforation) > stricturing > inflammatory. For consistency, similar categories were used for patients with UC.

**Table 1.** CT imaging findings in patients with Crohn’s disease and ulcerative colitis

Imaging finding	Crohn’s disease, *n* (%) (*n* = 1452)	Ulcerative colitis, *n* (%) (*n* = 418)
Normal	357 (24.59%)	82 (19.62%)
IBD findings		
Inflammation	337 (23.21%)	161 (38.52%)
Stricturing	345 (23.76%)	77 (18.42%)
Low-grade	60 (4.13%)	0 (0.00%)
High-grade (obstruction)	285 (19.63%)	77 (18.42%)
Penetrating	260 (17.90%)	32 (7.66%)
Fistula with obstruction	49 (3.38%)	1 (0.24%)
Fistula without obstruction	49 (3.38%)	4 (0.96%)
Phlegmon	26 (1.79%)	3 (0.72%)
Abscess	115 (7.92%)	20 (4.78%)
Perforation	21 (1.45%)	4 (0.96%)
*Inappropriate test	28 (1.93%)	0 0.00%
Non-IBD findings		
Luminal	22 (1.52%)	7 (1.68%)
Appendicitis	11 (0.76%)	2 (0.48%)
Diverticulitis	11 (0.76%)	5 (1.20%)
Extraluminal	103 (7.09%)	59 (14.11%)
Ureteric stone	65 (4.48%)	21 (5.02%)
Pancreatitis	11 (0.76%)	1 (0.24%)
Soft tissue infection	2 (0.14%)	1 (0.24%)
Venous Thromboembolism	2 (0.14%)	3 (0.72%)
Hepatobiliary	9 (0.62%)	9 (2.15%)
Varices	3 (0.21%)	0 (0.00%)
Gynecological	5 (0.34%)	8 (1.91%)
Hernia	5 (0.34%)	11 (2.63%)
Suspected malignancy	1 (0.07%)	5 (1.20%)

*Inappropriate diagnostic test = CT scans for isolated perianal disease.

The stricturing category was subdivided into low-grade and high-grade strictures/bowel obstruction. Obstruction required the CT report to comment on upstream bowel dilation or upstream small bowel diameter greater than 3 cm. In situations where multiple IBD and non-IBD findings were reported, only the dominant finding was recorded. When the results were not clear, the imaging study was reviewed with a senior radiologist and an interest in IBD imaging (B.M.).

Urgent IBD findings were defined as bowel obstruction, stricture with pre-stenotic dilation, phlegmon, abscess or perforation. Urgent penetrating findings were defined as phlegmon, abscess, or perforation. Non-urgent IBD findings included normal scans, inflammation only, strictures without obstruction and fistula without obstruction.

Finally, among patients who underwent a prior scan within a 1-year period, the scans for the two time points were compared and findings were categorized as improved, unchanged, worsened or new.

### Statistical Analysis

Descriptive statistics were used to report patient demographics, proportion of encounters that underwent CT imaging and CT findings. Categorical variables are presented as proportions, and continuous variables as means with standard deviation (SD) for normally distributed data or medians with interquartile range (IQR) for data not normally distributed.

Differences in CT utilization were assessed for IBD type, sex and age by Chi-squared test. For age, patients were stratified equally into quartiles. Results are expressed as relative risk (RR) with 95% CI.

Changes in the annual rates of CT utilization were assessed overall and for each disease type (CD, UC and IBDU) using a generalized linear model that assumed a Poisson distribution for the outcome CT utilization. Overdispersion assumption was assessed, and Pearson residual variance was found to approximate 1, suggesting no issues with overdispersion. Multiple subgroup analyses were performed for males, females, and by younger and older age based on the median age for each IBD subtype. Furthermore, we performed a sensitivity analysis restricting our cohort to patients with IBD (CD or UC) listed as the most responsible diagnosis. Results are expressed as annual change with 95% CI derived from the Poisson regression beta estimate.

Changes in the types of IBD findings over time were assessed using the Cochran-Armitage Trend Test or the Cochran-Mantel Haenszel Test where appropriate. For each analysis, statistical significance was considered positive when *P* < 0.05.

## RESULTS

### Patient Cohort

**Figure 1.**
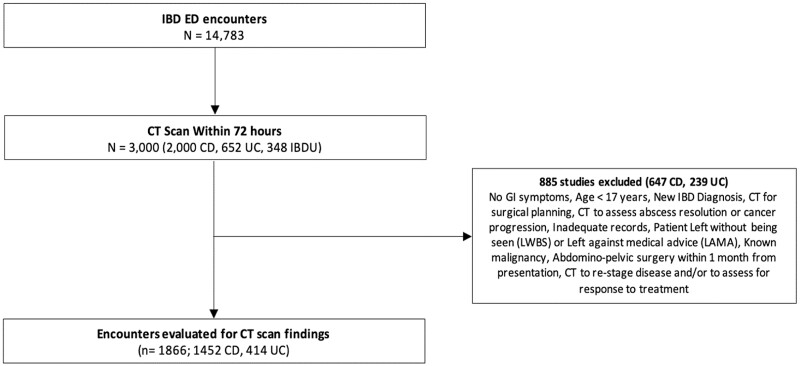
Study inclusion and exclusion criteria flow diagram.

A total of 14,783 ED encounters involving patients with an existing diagnosis of IBD were identified during our study period: 9132 with CD, 3946 with UC and 1705 with IBDU (Figure 1). The median age was 42 years (IQR 31 to 56), and 8079 encounters (55%) involved female patients. Among all encounters, the most responsible diagnosis was related to CD or UC in 2361 encounters (18%).

### CT Utilization

Overall, 3000 abdominopelvic CT scans were performed: 2000 for patients with CD (67%), 652 for patients with UC (22%) and 348 for patients with IBDU (12%) (Figure 1). This represented 22% of encounters for CD, 17% of encounters for UC and 20% of encounters for encounters for IBDU. Among encounters with IBD (CD or UC) listed as the most responsible diagnosis, abdominopelvic CT imaging was performed in 38% of encounters for CD, 21% of encounters for UC and 31% of encounters for IBDU. Younger patients were less likely to undergo CT imaging (Supplementary Table 2). Males with CD (RR, 1.21; 95% confidence interval [CI], 1.1 to 1.4; *P* = 0.0015), but not UC (RR, 1.15; 95% CI, 1.0 to 1.38; *P* = 0.14) or IBDU (RR, 1.18; 95% CI, 0.9 to 1.6; *P* = 0.26) were more likely to undergo CT imaging (Table 2).

**Table 2.** Annual increase in CT utilization stratified by age strata, sex and IBD subtype

Subgroup	Annual per cent increase (95% CI)	*P*-value
Crohn’s disease		
Female	4.56% (2.33–6.79%)	<0.0001
Male	3.44% (1.03–5.85%)	0.0053
Younger*	5.72% (3.08–8.36%)	<0.0001
Older^†^	2.36% (0.32–4.41%)	0.0234
Most responsible diagnosis^‡^	2% (−1.00–6.00%)	0.2100
Ulcerative colitis		
Female	4.50% (0.87–8.13%)	0.0152
Male	3.76% (−0.19–7.71%)	0.0619
Younger*	0.87% (−3.22–4.95%)	0.6766
Older^†^	5.30% (1.74–8.87%)	0.0036
Most responsible diagnosis^‡^	4.0% (−0.3–12%)	0.2000
Inflammatory bowel disease unclassifiable		
Female	4.75% (−1.42–10.92%)	0.1308
Male	8.11% (2.87–13.35%)	0.0025
Younger*	9.01% (1.90–16.12%)	0.0132
Older^†^	3.51% (−1.15–8.18%)	0.1392
Most responsible diagnosis^‡^	10.00% (0.1–21%)	0.0400

CI, confidence interval.

*Subgroup analysis of patients below median age.

^†^Subgroup analysis of patients above median age.

^‡^Sensitivity analysis of patients with IBD as most responsible diagnosis.

**Figure 2.**
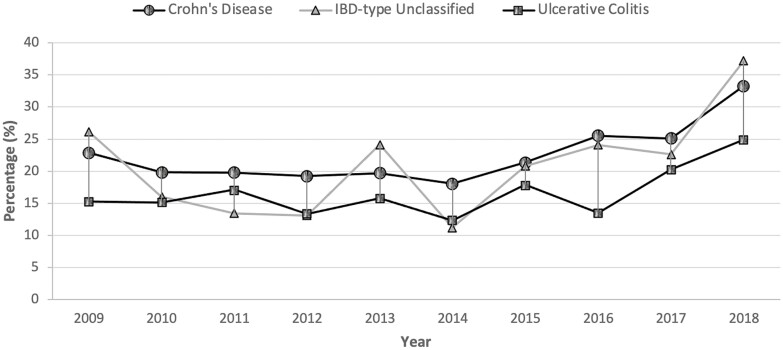
Annual abdominopelvic CT scan utilization among patients with Crohn’s disease, ulcerative colitis and inflammatory bowel disease unclassifiable who presented to the emergency department irrespective of the indication.

On average, CT utilization increased annually for each IBD subtype: by 2.7% (95% CI, 1.2 to 4.3; *P* = 0.0004) in CD, 4.2% (95% CI, 1.7 to 6.7; *P* = 0.0009) in UC and 6.3% (95% CI, 2.5 to 10.0; *P* = 0.0011) in IBDU (Figure 2). Compared to before 2016, CT utilization was greater after 2016 in CD (RR, 1.28; 95% CI, 1.2 to 1.4; *P* < 0.0001), UC (RR, 1.48; 95% CI, 1.3 to 1.7; *P* < 0.0001) and IBDU (RR, 1.54; 95% CI, 1.2 to 1.9; *P* = 0.0001). The annual percentage change in CT utilization was similar when encounters were stratified by age, sex, and when limiting our cohort to patients with IBD (CD or UC) listed as the most responsible diagnosis (Table 2).

### Characterization of CT Findings

A total of 1866 (62%) CT studies met our eligibility criteria for CT scan analysis: 1452 (78%) CD encounters and 418 (22%) UC encounters. CT studies were more likely to be reported as normal or contain inflammation alone in patients with UC compared with CD (58% vs. 48%; *P* < 0.001). In contrast, patients with CD were more likely to have obstructive complications (24% vs. 18%; *P* = 0.021) and penetrating complications (18% vs. 8%; *P* < 0.001). Urgent IBD findings (high-grade strictures, obstruction, phlegmon, abscess or perforation) were more common in patients with CD (31% vs. 25%; *P* = 0.015), of which 162 (11%) were urgent penetrating findings (phlegmon, abscess or perforation). Non-IBD findings were less common in patients with CD compared to patients with UC (9% vs. 16%; *P* < 0.001). The most common non-IBD findings in both cohorts were ureteric stones (Table 1).

### Trends in CT Findings Over Time

**Figure 3.**
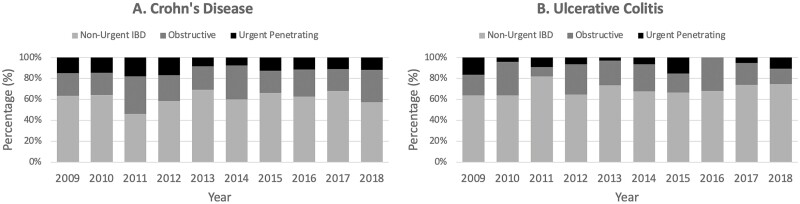
Annualized rates of CT findings in patients with (A) Crohn’s disease and (B) ulcerative colitis. Non-urgent IBD findings included those that were: normal, inflammation-only, stricture without obstruction, fistula without obstruction. Obstructive findings included: stricture with obstruction, fistula with obstruction. Urgent penetrating findings included: phlegmon, abscess, perforation.

Annual CT findings are reported in Table 3. The annual proportion of scans with urgent penetrating findings ranged from 7% to 16% in patients with CD and 0% to 13% in patients with UC. The annual proportion of scans with high-grade obstructive findings ranged from 19% to 32% in patients with CD and 9% to 29% in patients with UC. There were no significant changes in the CT findings over time for patients with CD (*P* = 0.13) or patients with UC (*P* = 0.17; Figure 3).

### Analysis of Repeat Imaging Within 1 Year

Of the 1452 imaging studies performed in patients with CD, 699 (48%) had a prior scan within the previous year. Compared to earlier scan, the majority (*n* = 421; 60%) remained unchanged or improved, while 278 (40%) demonstrated worsening disease or a new complication (Tables 3 and 4).

**Table 3.** Annualized proportion of abdominal CT scans with positive findings in patients with Crohn’s disease and ulcerative colitis

Crohn’s disease	2009 (*n* = 114)	2010 (*n* = 128)	2011 (*n* = 119)	2012 (*n* = 126)	2013 (*n* = 160)	2014 (*n* = 128)	2015 (*n* = 136)	2016 (*n* = 168)	2017 (*n* = 171)	2018 (*n* = 202)
Normal	29 (25%)	25 (20%)	21 (18%)	38 (30%)	40 (25%)	23 (18%)	34 (25%)	44 (26%)	54 (32%)	49 (24%)
Inflammation	25 (22%)	41 (32%)	18 (15%)	24 (19%)	45 (28%)	34 (27%)	33 (24%)	38 (23%)	43 (25%)	36 (18%)
Stricture	23 (20%)	25 (20%)	44 (37%)	28 (22%)	32 (20%)	37 (29%)	30 (22%)	38 (23%)	35 (20%)	53 (26%)
^†^Low-Grade	5 (4%)	3 (2%)	9 (8%)	3 (2%)	6 (4%)	5 (4%)	9 (7%)	4 (2%)	7 (4%)	9 (4%)
^‡^High-Grade	18 (16%)	22 (17%)	35 (29%)	25 (20%)	26 (16%)	32 (25%)	21 (15%)	34 (20%)	28 (16%)	44 (22%)
^*^Penetrating	24 (21%)	26 (20%)	23 (19%)	28 (22%)	26 (16%)	23 (18%)	21 (15%)	30 (18%)	22 (13%)	37 (18%)
Fistula without Obstruction	5 (4%)	6 (5%)	1 (1%)	4 (3%)	8 (5%)	8 (6%)	2 (1%)	8 (5%)	1 (1%)	6 (3%)
Fistula with Obstruction	4 (4%)	3 (2%)	3 (3%)	4 (3%)	6 (4%)	6 (5%)	4 (3%)	5 (3%)	4 (2%)	10 (5%)
^§^Urgent Penetrating	15 (13%)	17 (13%)	19 (16%)	20 (16%)	12 (8%)	9 (7%)	15 (11%)	17 (10%)	17 (10%)	21 (10%)
^‖^Non-IBD Luminal	1 (1%)	0 (0%)	4 (3%)	1 (1%)	2 (1%)	3 (2%)	4 (3%)	4 (2%)	1 (1%)	2 (1%)
^¶^Non-IBD Extraluminal	9 (21%)	10 (8%)	8 (7%)	4 (3%)	13 (8%)	6 (5%)	10 (7%)	12 (7%)	14 (8%)	17 (8%)
^#^Inappropriate Study	3 (3%)	1 (1%)	1 (1%)	3 (2%)	2 (1%)	2 (2%)	4 (3%)	2 (1%)	2 (1%)	8 (4%)
Ulcerative colitis	2009 (*n* = 51)	2010 (*n* = 28)	2011 (*n* = 35)	2012 (*n* = 41)	2013 (*n* = 43)	2014 (*n* = 38)	2015 (*n* = 39)	2016 (*n* = 50)	2017 (*n* = 46)	2018 (*n* = 57)
Normal	8 (16%)	5 (18%)	8 (23%)	6 (15%)	7 (16%)	6 (16%)	8 (21%)	12 (24%)	11 (24%)	11 (19%)
Inflammation	15 (29%)	11 (39%)	18 (51%)	14 (34%)	18 (42%)	14 (37%)	14 (36%)	18 (36%)	17 (37%)	22 (39%)
Stricture	7 (14%)	8 (29%)	3 (9%)	9 (22%)	8 (19%)	8 (21%)	5 (13%)	14 (28%)	8 (17%)	7 (12%)
^†^Low-Grade	0 (0%)	0 (0%)	0 (0%)	0 (0%)	0 (0%)	0 (0%)	0 (0%)	0 (0%)	0 (0%)	0 (0%)
^‡^High-Grade	7 (14%)	8 (29%)	3 (9%)	9 (22%)	8 (19%)	8 (21%)	5 (13%)	14 (28%)	8 (17%)	7 (12%)
^*^Penetrating	6 (12%)	1 (4%)	4 (11%)	2 (5%)	1 (2%)	3 (8%)	6 (15%)	0 (0%)	2 (4%)	7 (12%)
Fistula without Obstruction	0 (0%)	0 (0%)	1 (3%)	0 (0%)	0 (0%)	1 (3%)	0 (0%)	0 (0%)	0 (0%)	2 (4%)
Fistula with Obstruction	0 (0%)	0 (0%)	0 (0%)	0 (0%)	0 (0%)	0 (0%)	1 (3%)	0 (0%)	0 (0%)	0 (0%)
^§^Urgent Penetrating	6 (12%)	1 (4%)	3 (9%)	2 (5%)	1 (2%)	2 (5%)	5 (13%)	0 (0%)	2 (4%)	5 (9%)
^‖^Non-IBD Luminal	1 (2%)	0 (0%)	0 (0%)	2 (5%)	0 (0%)	0 (0%)	0 (0%)	1 (2%)	1 (2%)	2 (4%)
^¶^Non-IBD Extraluminal	12 (24%)	3 (11%)	2 (6%)	8 (20%)	7 (16%)	6 (16%)	5 (13%)	4 (8%)	6 (13%)	6 (11%)
^#^Inappropriate Study	0 (0%)	0 (0%)	0 (0%)	0 (0%)	0 (0%)	0 (0%)	0 (0%)	0 (0%)	0 (0%)	0 (0%)

^†^Low-grade = stricture without signs of obstruction.

^‡^High-grade = stricture with signs of obstruction or pre-stenotic dilation > 3 cm.

^*^Penetrating = fistula with or without obstruction, phlegmon, abscess, perforation.

^§^Urgent Penetrating = perforation, abscess, phlegmon.

^‖^Non-IBD Luminal = diverticulitis, appendicitis.

^¶^Non-IBD Extraluminal = ureteric stone, pancreatitis, soft tissue infection, VTE**, liver/pancreatic/gall bladder disease, gynecologic issue, hernia, new malignancy.

^#^Inappropriate Study = CT studies ordered to investigate isolated perianal disease.

**Table 4.** Annualized CT findings for encounters with a prior cross-sectional imaging performed within the last year before presentation in patients with Chron’s disease and ulcerative colitis

Crohn’s disease	2009 (*n* = 57)	2010 (*n* = 47)	2011 (*n* = 51)	2012 (*n* = 59)	2013 (*n* = 66)	2014 (*n* = 64)	2015 (*n* = 73)	2016 (*n* = 94)	2017 (*n* = 89)	2018 (*n* = 99)	Total (*n* = 699)
Improved	8 (14%)	9 (19%)	12 (24%)	18 (31%)	10 (15%)	12 (19%)	18 (25%)	21 (22%)	16 (18%)	26 (26%)	150 (21%)
Unchanged	21 (37%)	19 (40%)	15 (29%)	20 (34%)	34 (52%)	25 (39%)	29 (40%)	34 (36%)	45 (51%)	29 (29%)	271 (39%)
Worse	7 (12%)	7 (15%)	6 (12%)	7 (12%)	8 (12%)	10 (16%)	9 (12%)	11 (12%)	8 (9%)	20 (20%)	93 (13%)
New complication	21 (37%)	12 (26%)	18 (35%)	14 (24%)	14 (21%)	17 (27%)	17 (23%)	28 (30%)	20 (22%)	24 (24%)	185 (26%)
Ulcerative colitis	2009 (*n* = 11)	2010 (*n* = 8)	2011 (*n* = 9)	2012 (*n* = 12)	2013 (*n* = 15)	2014 (*n* = 16)	2015 (*n* = 16)	2016 (*n* = 24)	2017 (*n* = 16)	2018 (*n* = 24)	Total (*n* = 151)
Improved	2 (18%)	2 (25%)	3 (33%)	3 (25%)	3 (20%)	3 (19%)	0 (0.0%)	5 (21%)	2 (13%)	4 (17%)	27 (18%)
Unchanged	4 (36%)	2 (25%)	3 (33%)	3 (25%)	2 (13%)	4 (25%)	6 (38%)	10 (42%)	6 (38%)	9 (38%)	49 (32%)
Worse	5 (45%)	4 (50%)	3 (33%)	6 (50%)	10 (67%)	9 (56%)	10 (63%)	9 (38%)	8 (50%)	11 (46%)	75 (50%)
New complication	0 (0%)	0 (0%)	0 (0%)	0 (0%)	0 (0%)	0 (0%)	0 (0%)	0 (0%)	0 (0%)	0 (0%)	0 (0%)

Of the 418 imaging studies performed in patients with UC, 151 (36%) had a prior scan within the previous year. Compared to earlier scan, 76 (50%) remained unchanged or improved, while 75 (50%) demonstrated worsening disease or a new complication (Table 3).

## DISCUSSION

In this observational study, we demonstrated persistently high rates of CT utilization, with a modest increase over time, among patients with IBD who presented to the ED over the last decade. The increase in CT utilization was most pronounced after 2016. By the final year of our study, 60% of patients with CD and 33% of patients with UC with gastrointestinal symptoms underwent CT imaging. Less than one third of CT studies reported urgent IBD findings (high-grade strictures, obstructions, phlegmon, abscess or perforation) and a minority of CT studies reported urgent penetrating findings (phlegmon, abscess or perforation) (11% in CD and 6% in UC).

The rates of CT utilization in our study were higher than those reported in a tertiary care centre from Manitoba, Canada, between 2009 and 2011 (49% of patients with CD and 19% of patients with UC). However, findings were comparable to those reported in a tertiary care centre in Pennsylvania, USA between 2001 and 2009 (47% to 78% of patients with CD) (27,30). Unlike previous studies we observed, there was only a modest increase in CT utilization over the past decade (6,27,28). For example, Kerner and colleagues demonstrated an increase in the rate of CT imaging from 47% in 2001 to 78% in 2009 among patients with CD (27). Despite our noted modest increase in CT utilization, the distribution of CT findings remained stable over time, suggesting similar practice patterns over the last decade.

Recent studies have demonstrated reductions in overall CT utilization among the general population presenting to the ED with abdominal pain (31). The lack of reduction in CT imaging among our IBD population was somewhat unexpected given these findings, along with the recent Choosing Wisely guidelines (19) and expert consensus guidelines (20–26) recommending judicious use of CT imaging in the ED. However, the end of our study period was likely too close to the establishment of these guidelines to make formal conclusions about trends. Furthermore, it is important to note that the existing guidelines for patients with IBD were created by gastroenterologists and radiologists and may not address the specific practice needs of ED physicians and surgeons.

The decision to perform CT imaging in the ED requires careful balance of the potential risks and benefits with the theoretical risk of malignancy and health care resources impact. Although we found that a substantial proportion of scans for patients with CD had abnormal findings, similar to previous studies (27,30,32), it is important to recognize that only 11% of CT scans reported urgent penetrating findings (phlegmon, abscess, perforation). These complications are the most relevant for CT imaging, as they cannot be reliably diagnosed clinically, nor by plain radiography. In contrast, a clinical history alone or plain film imaging is often sufficient to diagnose obstructions (33–35). Finally, it should be noted that advances in CT imaging protocols and post-imaging processing techniques have reduced the effective dose of radiation per imaging study (36–38). Nevertheless, alternate safer imaging modalities exist such as abdominal ultrasound, which has been shown to be effective in detecting IBD complications (20–22,26,39–41). Although point of care ultrasound is used commonly in the ED for many clinical indications, it is not yet routinely used for IBD (42,43).

Interestingly, we found higher rates of urgent CT findings among our cohort with UC than what has been reported in previous studies (30,44,45). Caution is required when comparing studies, since past studies contained small numbers of patients (*n* = 23 to 132). Nevertheless, our exclusion of first-presentation IBD, where patients would be less likely to have complications, may partly explain why our study contained higher rates of urgent findings. It is also possible that our 72-h time period captured more patients who were admitted to hospital for complications compared to other studies. Finally, differences in known risk factors for disease complications including disease severity, corticosteroid exposure, and the proportion of patients who previously underwent colectomy, could also explain the discrepancy between studies (45).

Judicious use of CT imaging may have additional benefits including, reductions in health care costs, improving timely access to imaging, and improving patient flow in the ED. For these reasons, strategies to identify patients who are most likely to benefit from CT imaging are required. Several clinical prediction models have been created to identify patients at highest risk of having urgent findings (44,46–49). These models have reasonable performance characteristics but have not been prospectively validated.

To our knowledge, this is the first study to assess CT utilization in the ED since publication of the Choosing Wisely guidelines and societal position statements on the use of CT imaging in the ED (19–21,26). Our large sample size and study follow-up over a 10-year period allowed us to establish accurate, longitudinal estimates of CT utilization in our patient population. We also used a well-defined population with a pre-existing diagnosis of IBD. While this was important for determining practice patterns in patients with an established diagnosis of IBD, it likely resulted in an underestimate of CT utilization overall since resource utilization is highest at the time of diagnosis (50,51).

Our study had a number of important limitations. First, it was performed at a single academic centre and may not reflect the practice patterns of community EDs and other academic centres. Second, our liberal 72-h time-period to determine CT utilization may have captured imaging studies performed after discharge from the ED. However, these were likely a minority and would have represented the intent of the ED encounter. Third, the use of administrative data precluded us from determining the provider who ordered the CT scans to determine whether there were changes in practice patterns between specialties, or the time of day the scans were ordered to determine if the time of admission impacted CT utilization. Similarly, we were not able to determine disease severity to determine if the changes in CT utilization in our study were the result of changes in the severity/complexity of IBD patient’s overtime. Furthermore, our administrative codes for CT imaging have not been validated. Although scans that were not captured by our administrative codes may have resulted in an underestimate of CT utilization, there were no false positive scans as confirmed by our manual chart review. Fourth, we did not determine if the imaging studies had an impact on disease management. Although a previous study demonstrated that CT imaging led to a change in management in 81% of patients with CD and 69% of patients with UC, this can be subjective and difficult to establish in a retrospective study (30). Finally, we included all CT scans irrespective of the use/mode of delivery of contrast. The inclusion/exclusion of contrast may have impacted the diagnostic yield of individual CT studies.

## CONCLUSION

In conclusion, our single-centre study demonstrated persistently high rates of CT imaging in the ED among patients with IBD. This suggests that guidelines and recent expert opinion publications have not been sufficient to alter practice patterns. Future work is needed to evaluate clinicians’ perceptions on the value of CT imaging in the ED, determine major drivers influencing decisions of when to perform CT imaging, determine which clinical presentations are most likely to benefit from CT imaging, and to develop multidisciplinary practice guidelines applicable to all stakeholders (patients, ED physicians, surgeons, gastroenterologists and radiologists).

## Supplementary Material

gwac029_suppl_Supplementary_Tables
