# Supplementary material for: Persistently High Rates of Abdominal Computed Tomography Imaging Among Patients With Inflammatory Bowel Disease Who Present to the Emergency Department
Source: J Can Assoc Gastroenterol. 2022 Oct 28;6(2):64–72. doi: 10.1093/jcag/gwac029 (PMC10071298; doi:10.1093/jcag/gwac029)
Supplement: gwac029_suppl_Supplementary_Tables [file gwac029_suppl_supplementary_tables.docx]

**Supplementary Table 1:** Institutional CT codes used to identify abdominopelvic CT scans

**
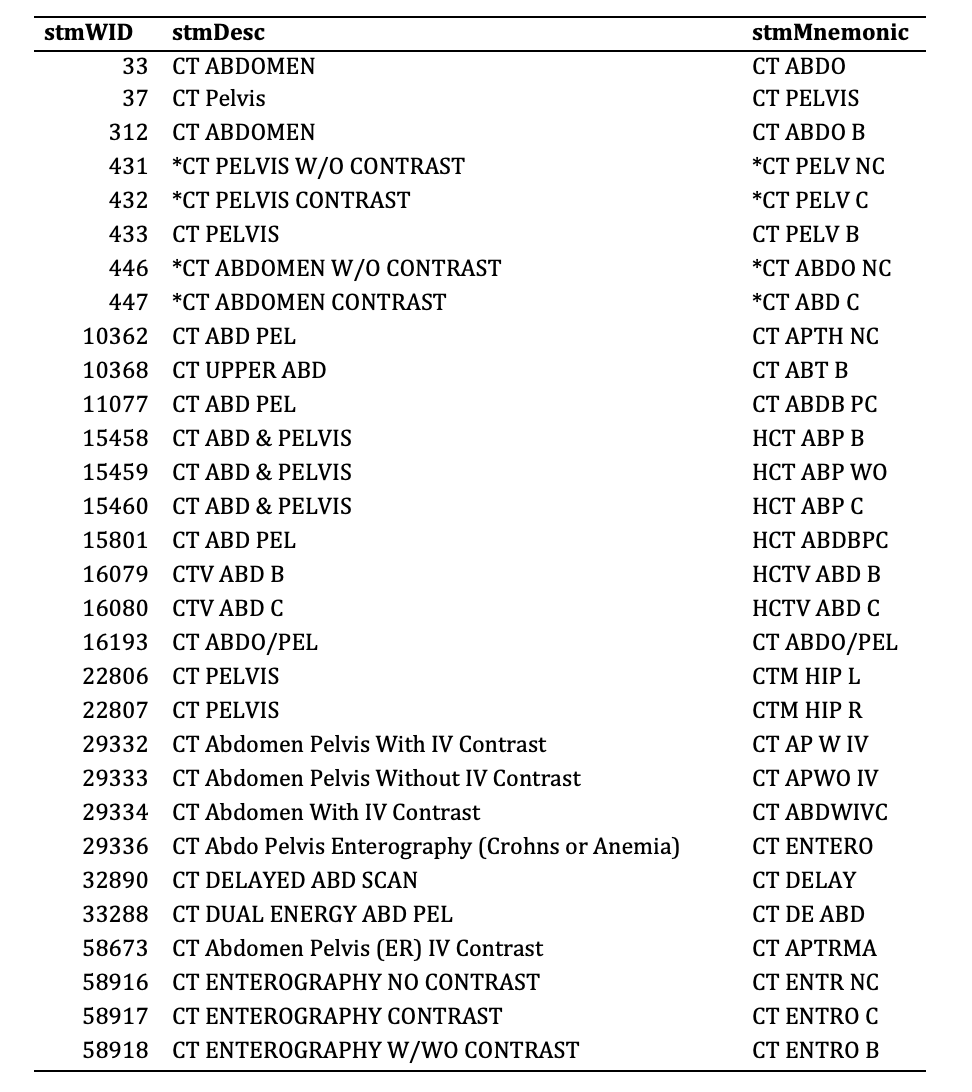
**

**Supplementary Table 2:** Relative risk of CT Scan exposure based on age and stratified by IBD Subtype.

*
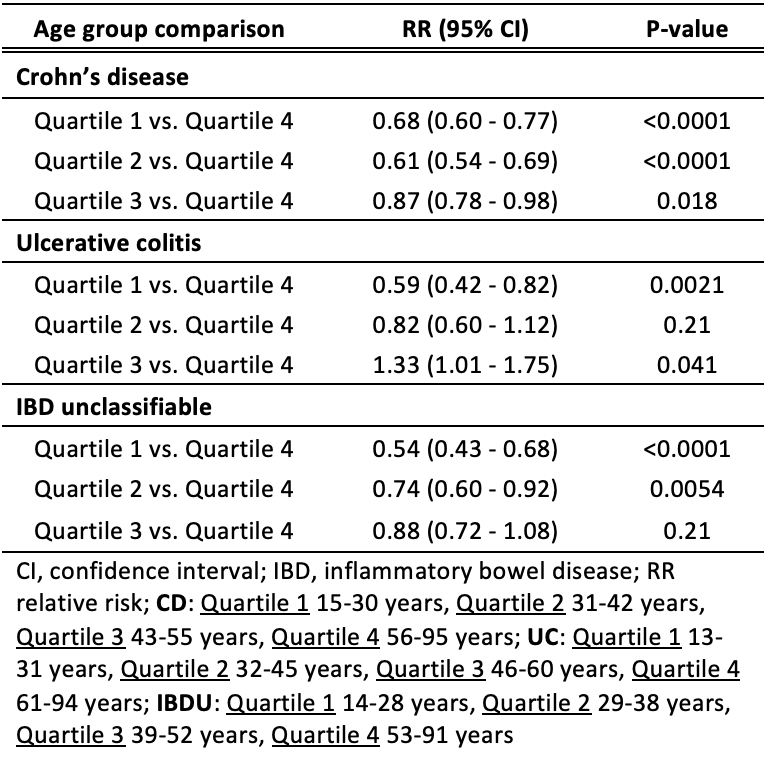
*
